# Retraction: The role of clustering algorithm-based big data processing in information economy development

**DOI:** 10.1371/journal.pone.0290863

**Published:** 2023-08-24

**Authors:** The PLOS ONE Editors

The *PLOS ONE* Editors retract this article [1] because it was identified as one of a series of submissions for which we have concerns about peer review integrity and similarities across articles. These concerns call into question the validity and provenance of the reported results. We regret that the issues were not identified prior to the article’s publication.

HM either did not respond directly or could not be reached.

## References

[1] Ma H (2021) The role of clustering algorithm-based big data processing in information economy development. PLoS ONE 16(3): e0246718. 10.1371/journal.pone.0246718 33705391 PMC7951923

